# Comparisons of the QLICP-HN and FACT-H&N instruments for measuring quality of life in patients with head and neck cancer

**DOI:** 10.3389/fonc.2025.1606655

**Published:** 2025-06-26

**Authors:** Fan Shen, Wenhua Chi, Xizi Yang, Gaofeng Li, Jianfeng Tan, Chonghua Wan

**Affiliations:** ^1^ School of Humanities and Management, Research Center for Quality of Life and Applied Psychology, Guangdong Medical University, Dongguan, China; ^2^ College of Nursing, Shaanxi University of Chinese Medicine, Xianyang, China; ^3^ Department of Psychology, Kunming Medical University Haiyuan College, Kunming, Yunnan, China; ^4^ Department of Thoracic Surgery, The Third Affiliated Hospital of Kunming Medical University, Kunming, China

**Keywords:** QLICP-HN, head and neck cancer, quality-of-life, FACT-H&N, instruments

## Abstract

**Background:**

Two head and neck cancer quality-of-life(QoL) measurement tools, the Functional Assessment of Cancer Therapy-Head and Neck (FACT-H&N) and the Quality of Life Instruments for Cancer Patients-Head and Neck Cancer (QLICP-HN), are widely used in China, but several researchers tend to be confused about which QoL measurement tool to choose before conducting QoL measurements. This investigation aimed to employ data procured from patients diagnosed with head and neck cancer to conduct a comparative analysis of these two assessment tools.

**Methods:**

Questionnaire outcomes were scrutinized at the subscale level by utilizing scale measurement analytics, correlation evaluation, validation examination, and association analyses.

**Results:**

Correlations between the two QoL instruments: the QLICP-HN and the FACT-H&N, fluctuated from r = 0.30 (indicating weak agreement) within the social/family domain to r = 0.80 (indicating robust agreement) within the psychological domain. Intermediate r values were associated with the remaining domains. Examination of typical correlations between the two subscales unveiled a moderate overall concurrence between the two tools (first typical correlation coefficient r = 0.89, although the overall redundancy remained at less than 57%). In the overall measurement performance, each of the two QoL tools exhibited particular strengths. However, the QLICP-HN showcased higher total scale internal consistency coefficients and a more extensive range of subscale internal consistency coefficients than the FACT-H&N scales, albeit it exhibited inferior discriminant and convergent validity.

**Conclusion:**

This empirical investigation highlights that, despite some overlap in the information provided by the two QoL instruments, substantial differences persist, thereby negating the possibility of one tool substituting for the other. Consequently, outcomes derived from these two QoL measures cannot be directly juxtaposed.

## Introduction

Quality of Life (QoL) is defined by the World Health Organization (WHO) as “individuals’ perception of their position in life within the context of their culture and value system, and in relation to their goals, expectations, standards, and concerns” (1). It is a multifaceted concept, intrinsically influenced by various factors, including physical health, psychological well-being, level of independence, social relationships, personal beliefs, and their interaction with pertinent environmental features. Significant advancements have been made in the treatment of malignant tumors; however, the efficacy of these treatments remains limited since most treatment modalities are largely palliative and aimed at mitigating patient suffering and enhancing QoL (2–4). The modern clinical paradigm has transitioned from a strictly biological to a biopsychosocial model, thereby making the QoL consideration in head and neck oncology a universally accepted necessity (5).

Patients are often grappling with a potentially fatal pathology, which means they must learn to adapt to the disease’s impact and how the subsequent treatment affects their appearance and basic abilities, such as swallowing, breathing, speaking, and their overall daily lives (6, 7). These patients often face physical impairment, economic hardship, and weakened social networks due to their inability to work. The implementation of QoL as a benchmark to evaluate the treatment outcomes for oncology patients is more responsive to the patient’s subjective perceptions and enhances the evaluation of treatment outcomes (8). As a subjective metric, QoL is frequently susceptible to various confounding factors when assessed at an individual level. Establishing an objective method to assess the QoL of cancer patients is a prerequisite for future advancements and a significant reference point for evaluating the effectiveness of treatment protocols (9). Consequently, a range of questionnaires that assess the personal QoL of cancer patients have evolved into practical tools.

Seven notable head and neck cancer-specific scales include UWQoL (10), EORTCQLQ-C30/H&N35 (11), FACT-H&N (12), PSS-HN (13), HNQoL (14), HNCI (15, 16), and QLICP-HN (17). Interestingly, the QLICP-HN, a personal QoL measurement scale, is often the preferred choice among domestic researchers conducting studies on the personal QoL of head and neck cancer patients. Yang et al. provided an evaluation of the reliability and validity of this scale when utilized on Chinese head and neck cancer patients (17). The Reality translation project stems from a global interest in applying the FACT framework among international scholars (18). Bonomi et al. presented the pre-translation and pretesting results of several Truth scales from English to Dutch, French, Italian, Norwegian, and Swedish (19).

The QLICP-HN could be considered the Chinese counterpart of the FACT-H&N measurement tool. Both QoL assessment instruments adhere to the modular setup concept, incorporating a generic core module and a specific module. There have been numerous articles comparing both national and international personal QoL measurement tools for cancer patients, which have yielded positive results. For instance, a review by Varni et al. examined the reliability and validity of the Pediatric Personal Quality of Life scale (PedsQL), the General Core scale, the Multidimensional Fatigue scale, and the Cancer Module (20). After comparing and evaluating the European Organization for Research and Treatment of Cancer Stomach Questionnaire (EORTC QLQ-STO22) and the Functional Assessment of Treatment in Gastric Cancer (FACT-GA), Woo A et al. found that both tools exhibited a strong internal consistency, retest reliability, sensitivity to change, and construct validity (21). Both questionnaires have been internationally validated with large numbers of patients undergoing various treatments, thereby demonstrating their versatile applicability. Giga L et al. used the International Classification of Functioning, Disability, and Health (ICF) to identify the most frequently used tools for assessing functional status in brain cancer patients and to compare their content and psychometric properties (22).

However, there is a paucity of comparisons between quality-of-life measurement scales specifically for patients with head and neck cancer. This establishes a compelling rationale for comparing updated versions of these two instruments, QLICP-HN(V2.0) and the FACT-H&N(V4.0). This study aimed to conduct a comparative analysis of these two QoL measurement tools using original data collected by administering both the QLICP-HN(V2.0) and the FACT-H&N(V4.0) to the same head and neck cancer patients (For the sake of simplicity, the version number is omitted later). The investigation was centered on three main research questions: (1) Do corresponding subscales of the two QoL measurement tools (e.g., the physical functioning subscale of the QLICP-HN and the physical health subscale of the FACT-H&N) assess the same aspects? If question one is answered negatively for at least one subscale, it becomes critical to explore the second question. (2) Do the two QoL measurement tools cover the same content, or are there aspects only addressed by one instrument? (3) Are psychometric properties, such as reliability and validity indicators, similar across both QoL instruments?

To address these questions, a variety of statistical methods were employed to illustrate the results. Thus, the aim of this study was to provide valuable insight for researchers in determining which of these two QoL measurement tools should be adopted in future research.

## Patients and methods

### Study population

The study involved patients diagnosed with primary head and neck cancer, who underwent clinical evaluation and treatment at the Affiliated Hospital of Guangdong Medical University. The inclusion criteria were as follows: (1) Participants should have been diagnosed with primary head and neck cancer, with no additional underlying diseases or cancers; (2) they must possess sufficient reading skills, enabling them to complete the survey independently; (3) participants needed to willingly agree to participate in the study, which was expressed through the completion of an informed consent form.

Out of the 135 patients that met the inclusion criteria, 100 patients successfully completed the 2 designated QoL surveys. This subset was included in the final data analysis. This sample size is consistent with the sample size used in previous similar studies that evaluated the quality of life of patients with head and neck cancer. Therefore, a sample size of 100 patients is deemed reasonable and feasible, as it ensures the reliability of the research results while also considering the resource and time constraints in actual research. All participants were appropriately informed about the study’s purpose and willingly provided their consent to participate.

### Survey tools

The two discussed QoL instruments form part of a broader questionnaire that also incorporates the evaluation of demographic and relevant disease-specific data. The two QoL instruments QLICP-HN and the FACT-H&N, are always administered in the same sequence, with the QLICP-HN preceding the FACT-H&N. **Table 1** delineates the primary attributes of both these instruments, which share similarities in scale length, response scale type, time frame, and subscale structure.

**Table 1:** Important features of the QLICP-HN and the FACT-H&N.

Characteristic	QLICP-HN		FACT-H&N	
	Domain/Facet	No. of Items	Domain	No. of Items
Total scale	5/14	46	5	38
Subscales	Physical	8	Physical	7
	Psychological	9	Emotional	6
	Social	8	Social/family	7
	Common symptoms and side effects	7	Functional	7
	Specific modules	14	Additional attention	11
Type of response scale	5-point ordinal scales	ALL	5-point ordinal scales	ALL
Time frame	Past week	ALL	Past week	ALL
Syntax structure of items	Question: ‘‘Did you…?’’	ALL	Statement: ‘‘1 am (feel, have)…’’	ALL

The QLICP-HN is one of the QoL instruments system called QLICP (Quality of Life Instruments for Cancer patients) developed by module approach (23–25). It comprises a general module (QLICP-GM) and a head and neck cancer-specific module. The QLICP-GM features 4 domains—physical functioning (8 items), psychological functioning (9 items), social functioning (8 items), and common symptoms and side effects (7 items)—totalling 32 items, arranged in 10 facets across these 4 domains (23). Meanwhile, the head and neck cancer-specific module encompasses 14 items, which are categorized into 4 facets. The complete scale encompasses 46 items, arranged in 14 facets, across 5 domains (or dimensions), each of which uses a 5-tiered scoring system. Each item is scored on a scale of 1 (not at all) to 5 (very much), while the scoring methodology for the scale involves a 5-point equidistant scale, where 1, 2, 3, 4, and 5 represent the levels of severity in sequential order. Items that indicate a better quality of life are scored as positive, whereas items representing a poorer quality of life are scored as negative. Positive items are scored directly, while negative items are computed as 6 minus the original score. The cumulative scores from each domain constitute the domain score, and the summation of these domain scores provides the overall score. The corresponding standard score (SS) for all domains and the overall were linearly converted to a 0–100 scale using the formula: SS=(RS-Min) ×100/R, where SS, RS, Min and R represent the standardized score, raw score, minimum score, and range of scores, respectively.

Similarly, the FACT-H&N is one of the Functional Assessment of Cancer Therapy (FACT) QoL tool system developed by Cella et al. (12). It comprises a general scale, also known as the FACT-G, which assesses the universal components for QoL in cancer patients and contains a specific module tailored for patients with head and neck cancer. The FACT-G consists of 27 items: 7 items pertaining to physical status (coded GP1-GP7), 7 to social/family status (coded GS1-GS7), 6 to emotional status (coded GE1-GE6), 7 to functional status (coded GF1-GF7), and an 11-item module specific to head and neck cancer (coded HN1-HN11) (26). For the scoring method, FACT-H&N adopts a 5-level scoring system, with each of the 38 items rated on a scale from 0 (not at all) to 4 (very much). Positive items are directly scored on a scale of 0 to 4, whereas negative items are reverse scored, whereby a higher response option implies poorer quality of life. The cumulative scores from each domain constitute the domain score, and the summation of these domain scores provides the overall score.

### Survey methodology

This study conducted a QLICP-HN questionnaire survey among participants prior to the FACT-H&N questionnaire survey. Maintain a 30-minute interval between two quality of life assessments. During the management period, participants are ensured to complete the questionnaire in a quiet and independent environment to minimize external interference and ensure the accuracy and reliability of the data.

The QLICP-HN is a self-evaluation tool, which necessitates a certain degree of literacy from the subjects to successfully complete the survey in a quiet and solitary environment. The purpose of the survey was clearly explained to all eligible participants before they began, emphasizing that participation was voluntary and that their responses would be kept confidential and cause no harm. After obtaining informed consent, the questionnaires were administered to the participants, and comprehensive instructions were provided to underline the importance of thoroughly answering each question. Then, the participants were asked to carefully respond to the questions. Subsequently, the completed questionnaires were collected and analyzed.

To assess test-retest reliability, 45 patients were selected to participate in the test-retest analysis. These 45 patients completed the second assessment within 2 days after the initial assessment. The time interval between the two assessments was 2 days. This interval was chosen to ensure that the health status of the patients remained relatively stable during this period, while also avoiding memory bias due to a shorter time span.

### Data analysis

The raw data were imported using EPIDATA 3.1, and a thorough analysis was performed using SPSS 26.0 statistical software. Subscores and total scores for the QLICP-HN and FACT-H&N were derived from the raw patient data. Additionally, it was necessary to compute the transformed FACT-H&N scores, converting the raw scores to a scale of 0–100 (where 0 is the lowest and 100 is the best quality of life), in a manner similar to the QLICP-HN processing.

For the first question—to determine whether the respective subscales of the QLICP-HN and the FACT-H&N measure similar facets—a correlation analysis was conducted. The study focused on the correlations between the corresponding subscales of these two QoL measurement instruments. The internal consistency of the subscales, as denoted by the Cronbach α coefficient, was used as a rough upper limit for the correlation r of the respective subscales (20, 27); hence, it served as a criterion to evaluate the consistency of the subscales (where a good consistency is indicated by r being approximately equal to α, and a poor consistency by r being substantially less than α). For descriptive purposes, the mean and standard deviation (SD) for the FACT-H&N and QLICP-HN subscales were calculated; even when subscales were converted to a common range of 0–100, it did not necessarily guarantee direct comparability; thus, formal statistical tests were avoided.

The second question—to determine whether the two QoL instruments collectively cover the same aspects or content—was addressed by calculating the typical correlation between the QLICP-HN and FACT-H&N subscale sets. This helped quantify the degree of overlap between the QLICP-HN and the FACT-H&N. The significance of the typical correlation was assessed using Bartlett’s method (27). The coefficient of redundancy (the fraction of variance in a subset of one instrument that can be ascribed to the typical variables of the other instrument) was employed as a measure of overlap between the two tools. If the two instruments cover the same content, the coefficient should be close to the mean of the square of the internal consistency α (or higher if the correlation between domains is substantial) (27).

For the third question—to determine whether the two QoL instruments are identical in terms of their measures of reliability and validity—an analysis was conducted to compare the measurement properties of the two distinct QoL instruments. This involved assessing the internal consistency coefficient, test-retest reliability, criterion-related validity, convergent validity, discriminant validity, responsiveness, and standardized response means.

Criterion-related validity was assessed by comparing the standardized scores of the two instruments. The correlation coefficient between the standardized scores of the QLICP-HN and FACT-H&N instruments was calculated to evaluate the consistency in measuring the same constructs. A correlation coefficient close to 1 indicates high criterion-related validity.

Convergent validity was assessed by evaluating the correlations between the subscales of the two instruments. The correlation coefficients between the corresponding subscales of the QLICP-HN and FACT-H&N instruments were calculated. Convergent validity was assessed using the average variance extracted (AVE) and composite reliability (CR), with AVE values greater than 0.5 and CR values greater than 0.7 indicating strong convergent validity.

Responsiveness was assessed by evaluating the ability of the two instruments to detect changes in patients’ quality of life. The standardized response means (SRMs) of the two instruments were calculated, with SRM values greater than 0.5 indicating strong responsiveness.

Standardized response means (SRMs) were calculated to assess the sensitivity of the two instruments to changes in patients’ quality of life. SRMs were calculated as the ratio of the mean change scores to the standard deviation of the change scores. Higher SRM values indicate greater sensitivity to change. SRMs were calculated for both the QLICP-HN and FACT-H&N instruments to evaluate their ability to detect changes in patients’ quality of life.

The potential impact of the scale measurement order (QLICP-HN preceding FACT-H&N) on the response levels was scrutinized. Scores on the nearly identical four QLICP-HN and FACT-H&N items were compared using a paired t-test (after linear conversion to the Generalized Response Scale).

### Ethics

All patients signed an informed consent form before participating in the study. The study was supported by the Affiliated Hospital of Guangdong Medical University

Ethics Committee (Opinion No. YJYS2019010) for approval.

## Results

### Demographic characteristics of participants

Regarding demographic characteristics (see **Table 2** in detail), the gender distribution was 41% male and 59% female. For ethnicity, 78% were Han Chinese and 22% were from other ethnic groups. Occupation-wise, 35% were farmers, 20% were workers, and the remaining 45% were in other occupations. Regarding family financial status, 64% reported a medium status, 32% reported poor status, and 4% reported good status. For medical treatment funding, 38% were covered by social medical insurance, 41% by cooperative medical treatment, and 21% were self-funded or had commercial medical insurance.

**Table 2:** The sample characteristics in patients with head and neck cancer.

Characteristics	N	%	Characteristics	N	%
Gender			Marital status		
Male	41	41	In marriage	95	95
Female	59	59	Unmarried	5	5
Ethnic group			Age		
Han Chinese	78	78	21-55	85	85
Other	22	22	55-71	15	15
Occupation			$\bar{X}\pm S$	44.44 ± 11.69	
Worker	20	20	Degree		
Farmer	35	35	Elementary	15	15
Other	45	45	Junior	25	25
Household financial situation			High school or secondary school	29	29
Poor	32	32	Junior college	17	17
Middle	64	64	Bachelor degree or above	14	14
Good	4	4			
Medical forms					
Social medical insurance	38	38			
Cooperative Healthcare	41	41			
Commercial medical insurance + self-pay	21	21			

### Scores comparisons between QLICP-HN and FACT-HN

**Table 3** presents the mean and SD for the subscale scores of the two QoL instruments. All FACT-H&N scores were converted to the conventional range of 0–100 (in alignment with the QLICP-HN) to enhance the clarity of the table. However, a direct comparison of the mean subscale scores from the two instruments may not be valid, meaning the P-values for these comparisons are not reported. Nevertheless, it’s important to note the differential ranking of the subscale means for the QLICP-HN and FACT-H&N. Specifically, the mean score for physical functioning was the lowest among the subscales of the QLICP-HN scale (significantly lower than the scores for social functioning, concomitant symptoms, and aftereffects; Wilcoxon paired test, P < 0.01), while the differences in mean scores for the subscales of the FACT-H&N scale were not statistically significant and no meaning for a ranking of subscales (P > 0.1). This indicates that the QLICP-HN scale facilitates cross-sectional comparisons across subscales within the scale, a capability not shared by the FACT-H&N.

**Table 3:** Scores of domains of the QLICP-HN and FACT-H&N.

QLICP-HN				FACT-H&N			
Domains	Min	Max	Mean ± SD	Raw Score	Standardized score, 0-100		
					Min	Max	Mean ± SD
Physical	12.5	93.75	59.81 ± 16.97	18.99 ± 5.78	0	100	67.82 ± 20.66
Psychological	8.33	100	62.47 ± 20.06	15.88 ± 5.49	0	100	72.18 ± 24.96
Social/Family	37.5	93.75	72.69 ± 12.54	19.99 ± 4.51	0	100	71.40 ± 16.11
role/functional	14.29	100	76.39 ± 18.84	10.15 ± 5.42	0	100	33.89 ± 20.06
The specific module	37.5	98.21	75.38 ± 12.86	18.46 ± 5.28	0	100	63.65 ± 18.20
Total score	38.24	88.89	68.96 ± 12.86	83.47 ± 20.45	0	100	56.64 ± 19.48

When comparing the score characteristics between the two scales, it was observed that the minimum values for each domain in the FACT-H&N as well as the overall scores were smaller than for the QLICP-HN; the maximum values for each domain of the FACT-H&N as well as the overall scores were larger than for the QLICP-HN, with the exception of the role functioning domain. Several QLICP-HN scale entries (GSS3, GPH6, GSO1, ADD4, SNH7, SNH10, and SNH11) exhibited a ceiling effect; entries of the FACT-H&N scale (GE3 and GE5) showed a ceiling effect and entries (HN8 and HN9) manifested a floor effect. This suggests that the QLICP-HN only possessed entries that demonstrate a ceiling effect, while the FACT-H&N contained entries that exhibited both a ceiling effect and a floor effect.

### Potential impact of the order

Four items from the two QoL measurement questionnaires were selected and assessed on an almost identical scale. For comparative purposes, the scores of these 4 items from the 2 questionnaires were linearly transformed to fall within the 0–10 range. Significant differences (p < 0.01; paired t-test) were observed in all 4 items: pain (QLICP-HN: 3.79 ± 1.20; FACT-H&N: 2.79 ± 1.14), sadness/grief (QLICP-HN: 3.67 ± 1.28; FACT-HN: 2.92 ± 1.18), sleep quality (QLICP-HN: 2.58 ± 0.91; FACT-H&N: 1.22 ± 1.01), and nausea/vomiting (QLICP-HN: 4.31 ± 0.99; FACT-H&N: 3.28 ± 1.12). Thus, the statistical results imply systematic variation in the response levels between the QLICP-HN (completed first) and the FACT-H&N (completed second) tools.

### Correlations among the QLICP-HN and FACT-H&N subscales

The study aimed to ascertain whether the corresponding subscales of the two QoL instruments measured similar constructs. Pearson correlations were calculated to analyze the relationship between the subscales of these two QoL measurement instruments, as depicted in **Table 4**. Notably, if two subscales measure analogous aspects, their correlation should closely align with the internal consistency of the two scales (α). Thus, by applying this criterion, only two pairs of the corresponding subscales demonstrated strong correlations: the physical functioning subscale (r = 0.723, compared to α = 0.805 and α = 0.98), and the emotion subscale (r = 0.804, compared to α = 0.864 and α = 0.994). For the concomitant symptoms (r = 0.504, compared to α = 0.85 and α = 0.993) and the specificity module subscale (r = 0.557, compared to α = 0.75 and α = 0.982); however, the correlations were less than anticipated. For the social/role functioning domain (r = 0.307, compared to α = 0.618 and α = 0.815), the correlations were also notably low. Collectively, the correlation analyses suggest that the study’s hypothesis (whereby the corresponding subscales of the QLICP-HN and the FACT-H&N could measure similar constructs) must be rejected. This is especially evident for the physical and emotion functioning subscale, and to a lesser extent, for the common symptoms and aftereffects, as well as the specific module areas.

**Table 4:** Pearson correlations between domains of the QLICP-HNand FACT-H&N.

QLICP-HN domains	FACT-H&N domains				
	Physical(α=0.98)	Social/family(α=0.815)	Emotional(α=0.994)	Functional(α=0.993)	Additional attention(α=0.982)
Physical,α=0.805	.723**	0.196	.677**	.658**	.644**
Psychological,α=0.864	.775**	0.182	.804**	.640**	.694**
Social,α=0.618	.640**	.307**	.588**	.528**	.491**
Common symptoms and side effects,α=0.85	.727**	.207*	.650**	.504**	.676**
Specific modules,α=0.75	.591**	.248*	.600**	.492**	.557**

**Significant correlation at 0.01 level (two-tailed).

*Significant correlation at 0.05 level (two-tailed).

The bold values indicate meaningful associations between the corresponding domains.

The complete correlation matrix is represented in **Table 4**. However, it is particularly noteworthy that for the QLICP-HN social/family, shared symptoms and side effects, and specific modules subscales, their correlations with their respective FACT-G counterparts were less than their correlations with other FACT-G subscales. This is another indicator of the lack of comparability between the two sets of subscales.

The research also investigated whether the two QoL instruments could comprehensively cover the same constructs, or whether each instrument covers unique and specific aspects. A crossover analysis (analyzing overt repetitiveness) between the FACT-H&N and QLICP-HN instruments was conducted using correlations for both corresponding subscales. This analysis yielded 2 statistically significant pairs of canonical variables (canonical correlations, r = 0.89 and r = 0.324; P < 0.05, Bartlett’s test). Further canonical correlations did not achieve statistical significance (P < 0.07). A high initial canonical correlation indicates a significant overlap between the two instruments in one dimension; however, for this dimension, exactly the first typical variable, showed strong, moderate, and weak correlations with the FACT-H&N social-family functioning subscale, physical subscale, affective subscale, functional status subscale, and the additional concerns subscale, respectively. However, for the QLICP-HN, it was strongly correlated with the psychological functioning subscale and weakly correlated with the remaining domains. Notwithstanding, the overall concurrence between these two multidimensional QoL measures was not substantial. Of the total variance for the QLICP-HN subscale, 56.3% could be accounted for by the full set of canonical variables in the FACT-H&N (yielding a redundancy factor of 0.563), and 46.9% of the total variance in the FACT-H&N subscale could be accounted for by the set of canonical variables in the QLICP-HN (a redundancy factor of 0.469). These proportions of variance must be contrasted with the proportions of variance anticipated when the same aspects are gauged by both instruments, i.e., 61.2% for QLICP-HN and 71.0% for FACT-H&N, as delineated in the Patients and Methods section. The discrepancy between the observed redundancy and the expected redundancy implies that the two instruments are not universally consistent.

### Psychometric properties of QLICP-HN and FACT-H&N

The validity and responsiveness of the two QoL assessment tools were shown to be consistent. The internal consistency coefficients of the QLICP-HN, along with the range of internal consistency coefficients of the subscales, were found to exceed those of the FACT-H&N. For the evaluation of convergent validity, the average variance extracted (AVE) and composite reliability (CR) were employed, with values greater than 0.5 for AVE and greater than 0.7 for CR generally indicative of strong convergent validity. The results demonstrated that the CR values for the FACT-H&N were all above 0.7 and that all the subscale AVE values, except for the social/family status subscale, were equal to or exceeded 0.5. Conversely, in the QLICP-HN, the AVE values for each subscale were below 0.5 and the CR values for each subscale were also below 0.7, suggesting weak convergent validity. From these observations, it can be concluded that the overall validity of the FACT-H&N scale surpasses that of the QLICP-HN.

An evaluation of the discriminant validity of the QLICP-HN demonstrated that for physical conditions, a square root of the AVE value of 0.593 was less than the maximum absolute value of the inter-factor correlation coefficient, which was 0.816. For social/family status, a square root of the AVE value of 0.549 was less than the maximum absolute value of the inter-factor correlation coefficient of 0.704. This pattern was maintained in the affective condition, where a square root of the AVE value of 0.671 was less than the maximum absolute inter-factor correlation coefficient value of 0.816. Similarly, in the functional condition, a square root of the AVE value of 0.687 was less than the maximum absolute inter-factor correlation coefficient value of 0.732. For the specific module, a square root of the AVE value of 0.485 was smaller than the maximum absolute value of the inter-factor correlation coefficient of 0.672. These results indicate poor performance in the discriminant validity for each scale in the QLICP-HN.

Contrastingly, the discriminant validity demonstrated more robust results for the FACT-H&N scale. For physical conditions, a square root of the AVE value of 0.796 exceeded the maximum absolute value of the inter-factor correlation coefficient of 0.730. In the social/family status category, a square root of the AVE value of 0.673 surpassed the maximum absolute value of the inter-factor correlation coefficient of 0.314. This pattern continued in the affective condition, where a square root of the AVE value of 0.798 was greater than the maximum absolute value of the inter-factor correlation coefficient of 0.746. For the functional condition, a square root of the AVE value of 0.789 exceeded the maximum absolute value of the inter-factor correlation coefficient of 0.636. The sole exception was found in the additional concerns (HNCS) category, where a square root of the AVE value of 0.553 was less than the maximum absolute value of the inter-factor correlation coefficient of 0.746.

These findings suggest good discriminant validity for each subscale in the FACT-H&N scale, except for the additional concerns subscale. The results also indicate that the FACT-H&N scale has superior discriminant validity than the QLICP-HN. For further details, please refer to **Tables 5** and **6**.

**Table 5:** Comparisons of psychometric properties of the QLICP-HN and FACT-H&N.

Psychometric properties	QLICP-HN	FACT-H&N
Test-retest reliability of each domain	0.983 -0.995	0.815 - 0.994
Test-retest reliability of the overall	0.994	0.977
Validity (correlation coefficient of the same/similar domain)	0.307 -0.804	0.307 -0.804
Validity (correlation coefficient of non-same/similar domain domain)	0.182 -0.775	0.182 - 0.775
Responsiveness (number of statistical domains/total domains)	4/5	4/5

**Table 6:** Convergent and discriminant validity of the QLICP-HNand FACT-H&N.

Domain	FACT-H&N				QLICP-HN			
	Average variance extracted AVE values	Combined confidence CR values	MSV value (maximum of shared squared variance)	ASV value (average of shared squared variance)	Average variance extracted AVE values	Combined confidence CR values	MSV value (maximum of shared squared variance)	ASV value (average of shared squared variance)
Physical	0.634	0.922	0.579	0.54	0.352	0.802	0.871	0.865
Social/family	0.453	0.816	0.081	-0.089	0.301	0.674	0.842	0.805
Emotional	0.636	0.908	0.832	0.509	0.45	0.875	0.871	0.865
role/functional	0.623	0.92	0.506	0.505	0.472	0.856	0.692	0.807
Specific modules	0.305	0.761	0.832	0.513	0.236	0.769	0.778	0.801

## Discussions

The QoL evaluation is gaining prominence as a comprehensive method for assessing health status and treatment outcomes. In the present study, analysis and evaluation were conducted on two distinct QoL assessment tools: the QLICP-HN and the FACT-H&N. The inquiry focused on whether these two discrete QoL assessment tools were distinctive or potentially interchangeable.

Three primary findings emerged from this research. Firstly, while both QoL tools address different facets of quality of life, there was some overlap in certain aspects. Secondly, although the subscales of the two QoL tools bear similar names, suggesting comparable meanings, only a few of these subscales contained consistent content. The content of the other subscales varied significantly, thereby indicating that the two tools are not directly comparable. The findings suggest discernible differences between the two QoL tools, indicating that they are not interchangeable. Consequently, the results derived from these two QoL tools cannot be directly compared, thereby underscoring the fact that they cannot be substituted for one another.

Further commonalities and differences can be observed in the structure and scoring characteristics of the scales. Both QoL tools employ a combination of core and specific modules, both employ a five-level graduated scale, and both encompass positive and negative items. However, the syntactic structure of the items in the FACT-H&N scale is declarative, while the syntax of the items in the QLICP-HN scale is interrogative (refer to **Table 1** for details).

Upon fully converting the scale to a generalized response scale, it was discovered that the range of extreme values for both the total score and the subscale scores of the FACT-H&N scale was larger than that of the QLICP-HN. This suggests that the FACT-H&N scale might encompass a broader measurement range and can potentially yield more precise QoL scores for some of the patients’ extreme values. Excluding the emotional and physical functioning subscales, the QLICP-HN scale demonstrated a more favorable performance than the FACT-H&N for all the remaining subscale scores and for the overall scale score (refer to **Table 2** for details). Moreover, a ceiling effect was identified for the entry into both the QoL tools, and a floor effect was observed for the entry into the FACT-H&N.

However, as depicted in **Table 4**, the physical functioning subscale in QLICP-HN exhibited significant and substantial correlations with its counterpart: the FACT-H&N physical functioning subscale. Furthermore, the QLICP-HN, within the emotion function scale and the co-occurring symptoms and side-effects scale, exhibited superior correlations with the FACT-H&N physical condition subscale. This might be attributed to the way the FACT-H&N physical functioning subscale partitions items into the physical condition subscale, placing items related to mental and co-occurring symptoms and side effects within the physical condition subscale.

Upon closer analysis, distinctions in the orientation of similarly named subscales were observed. It is also crucial to note that one of the two QoL instruments encapsulates specific aspects and dimensions that were either minimally or not at all covered by the other QoL instrument. The degree of overlap between these two QoL instruments was merely moderate and considerably lower than the anticipated level of overlap for measuring equivalent scales.

A critical aspect to acknowledge is that the selected participants predominantly consisted of Chinese individuals. Considering the influence of diverse religious beliefs, cultures, habits, and geographical locations on QoL, varying outcomes might arise if the selected participants were not Chinese. For instance, when Lu Q et al. (28) employed the FACT-B scale to compare QoL differences between US and Chinese breast cancer survivors, they discovered that Chinese breast cancer survivors who underwent chemotherapy reported significantly lower FACT-G scores than those who did not receive chemotherapy; a disparity that was not evident among US breast cancer survivors. Thus, this study also possesses a significant limitation, whereby it lacks diversification and internationalization in the sample source. Consequently, the conclusions drawn from the results of this study may primarily apply to the Chinese population of head and neck cancer patients.

Furthermore, the paired t-test identified statistical outcomes indicating systematic variation in response levels between QLICP-HN (completed first) and FACT-H&N (completed second). This suggests that the obtained QoL scores may differ depending on whether the QLICP-HN or the FACT-H&N was completed first. Thus, the sequence in which the scales are completed could potentially confound the results of the study. In addition, it should be noted that in this study, the QLICP-HN questionnaire was always conducted before the FACT-H&N questionnaire. This consistent testing sequence may introduce bias, as patients’ responses to the second questionnaire may be influenced by their experience with the first questionnaire. Future research should consider randomized questionnaire testing sequence or adopt single blind or double-blind design to mitigate this potential bias. When interpreting the research results, this limitation should be taken into account.

The critical question facing a potential user prepared to utilize both QoL assessment tools is determining the most suitable one for their requirements. Given the limitations of the aforementioned study, caution is necessary when providing advice. Essentially, the FACT-H&N tool appears to be more advantageous when the researcher’s objectives extend beyond the use of clinical settings, especially when socio–family status characteristics need to be incorporated. This is primarily because social, family and other characteristics are more likely to present extreme values. The broader range of these extreme values offered by FACT-H&N suggests that the patient’s QoL can be gauged more precisely, even in the presence of such extreme values.

Conversely, the QLICP-HN might be the optimal tool for clinical applications focusing on specific domains, such as symptoms and side-effects, rather than considering the impact of social and family factors on QoL. Moreover, this tool might be more appealing to clinicians who deal with advanced cases of head and neck cancer.

## Conclusion

Both the QLICP-HN and the FACT-H&N tools have their distinct advantages, demonstrating the prudence of having multiple QoL tools available concurrently. Thus, rather than striving to identify a ‘perfect’ QoL instrument, the appropriate choice should be made based on the specific research requirements and the characteristics of each QoL instrument at hand. Going forward, it is anticipated that these two QoL tools will continue to evolve and enhance their capabilities, borrowing strengths from each other, and continuing to enhance their improvements.

## Data Availability

The dataset is restricted for academic research purposes only and shall not be used for any commercial purposes. Requests to access these datasets should be directed to CW,wanchh@hotmail.com.
